# A Real-World Nationwide Study on COVID-19 Trend in Italy during the Autumn–Winter Season of 2020 (before Mass Vaccination) and 2021 (after Mass Vaccination) Integrated with a Retrospective Analysis of the Mortality Burden per Year

**DOI:** 10.3390/microorganisms12030435

**Published:** 2024-02-21

**Authors:** Luca Roncati, Carlo Galeazzi, Giulia Bartolacelli, Stefania Caramaschi

**Affiliations:** 1 Department of Surgery, Medicine, Dentistry and Morphological Sciences with Interest in Transplantation, Oncology and Regenerative Medicine, University of Modena and Reggio Emilia, 41121 Modena, Italy; 2 Department of Maternal, Infant and Adult Medical and Surgical Sciences, University of Modena and Reggio Emilia, 41121 Modena, Italy

**Keywords:** COVID-19, SARS-CoV-2, nucleoside-modified mRNA (modRNA), vaccination, vaccine-induced immune thrombotic thrombocytopenia (VITT), immunosenescence, inflammaging, older adults, lockdown, Italy

## Abstract

SARS-CoV-2 virulence is known to increase with lowering of environmental temperature and solar ultraviolet radiation; therefore, we have focused our real-world nationwide study concerning with COVID-19 trend and dynamics on the coldest seasons of the year in Italy, the Western country hardest hit at the onset of the pandemic, comparing the autumn–winter of 2020 (before mass vaccination but when the emergency machinery was fully operative in terms of tracing and swabs) with the autumn–winter of 2021 (after mass vaccination), and analyzing the mortality burden by age groups and life stages in the years 2019 (pre-COVID-19), 2020 (before mass vaccination), and 2021 (after mass vaccination). Methods: During the state of national health emergency, the Civil Defense Department released the aggregate data coming from the Higher Institute of Health, the Ministry of Health, the Italian Regions, and the Independent Provinces, to inform the population about the pandemic situation, daily. Among these data, there were the number of contagions, performed swabs, hospitalizations in Intensive Care Units (ICU), non-ICU patients, and deaths. By means of a team effort, we have collected and elaborated all these data, comparing the COVID-19 pandemic in Italy during the autumn–winter of 2020 with the autumn–winter of 2021. Moreover, we have extracted from the database of the National Institute of Statistics the total number of annual deaths in Italy during the years 2019, 2020, and 2021, comparing them to each other in order to evaluate the mortality burden attributable to COVID-19. Results: From the autumn–winter of 2020 to the autumn–winter of 2021, the contagions increased by ≈285%, against a ≈290% increase in the performed swabs; therefore, the mean positivity rate passed from 8.74% before mass vaccination to 8.59% after mass vaccination. The unprecedent vaccination campaign allowed a ≈251% abatement in COVID-19 deaths, and a reduction of ≈224% and ≈228% in daily ICU and non-ICU hospitalizations due to COVID-19, respectively. Regarding COVID-19 deaths, in 2020, there was a mortality excess of ≈14.3% quantifiable in 105,900 more deaths compared to 2019, the pre-COVID-19 year; 103,183 out of 105,900 deaths occurred in older adults (≥60 years), which is equivalent to ≈97.4%, while in adults over 50, the segment of population just below older adults, in 2020, there were 2807 more deaths than in 2019. Surprisingly, from the analysis of our data, it is emerged that in people under the age of 40 in the years 2019, 2020, and 2021, there were 7103, 6808, and 7165 deaths, respectively. This means that in subjects under 40 during 2020, there were 295 fewer deaths than in 2019, while during 2021, there were 357 more deaths than in 2020, equivalent to ≈5.2% more. Conclusions: COVID-19 is a potential life-threatening disease mainly in older adults, as they are the most vulnerable due to inherent immunosenescence and inflammaging. Extensive vaccination in this segment of population with up-to-date vaccines is the means to reduce deaths, hospitalizations, and ICU pressure in the public interest. In the event of future threats, a new mass vaccination campaign should not be implemented without taking into account the individual age; it should primarily be aimed at people over 60 and at patients of any age with immune deficits, and secondly at people over 50. COVID-19 vaccination shows a favorable benefit–risk ratio in older adults, while the balance steps down under the age of 40; this younger segment of the population should be therefore exempt from any mandatory vaccination.

## 1. Introduction

Since 4 May 2023, Coronavirus Disease 2019 (COVID-19) is an established and ongoing health issue which no longer constitutes a public health emergency of international concern. However, according to the World Health Organization (WHO), the next pandemic is a matter of ‘when,’ not ‘if’, and therefore we need to treasure past experiences to be better prepared for the future, with the awareness of having new technological platforms at our disposal, such as that based on nucleoside-modified mRNA (modRNA) [1].

It was 11 March 2020 when the WHO declared COVID-19 a pandemic; since then, there have been more than 750 million confirmed cases of COVID-19, including nearly 7 million deaths [2]. It represents the most dramatic pandemic of the new millennium, the viral infection with the highest media coverage in history, and a human disease that has put a strain on health, social and political systems, globally. Although it had been expected for years by insiders after the past outbreak of the Severe Acute Respiratory Syndrome (SARS), the world was not ready to face COVID-19; however, thanks to an extraordinary research effort, specific vaccines were discovered in record time, and made available on a large scale in early 2021 [1]. To date, more than 13 billion doses have been administered worldwide, of which almost 150 million were administered in Italy [2,3].

Originally identified in the Chinese city of Wuhan, Italy was the first Western country to be hit hard by COVID-19 and to experience lockdown restrictions under a democratic framework, which in 2019 consisted of 60.32 million people, then progressively decreased to 59.26 million in 2020, 58.98 million in 2021, and 58.85 million in 2022 [4,5,6,7].

Like all respiratory viruses, COVID-19 is influenced by environmental temperature and solar ultraviolet radiation; in fact, the virulence of the SARS Coronavirus 2 (SARS-CoV-2), its etiological agent, has been found to be maximum below 10 °C and 40 kJ/m^2^, respectively [8,9]. In Italy, these meteorological conditions are typical of the autumn–winter periods; therefore, we have focused our real-world nationwide study on these seasons.

Just over four years after that fateful 11 March 2020, let us take stock of the COVID-19 pandemic in Italy during the autumn–winter of 2020, before mass vaccination but when the emergency machinery was fully operative in terms of tracing and swabs, comparing this period with the autumn–winter of 2021, i.e., after the mass vaccination campaign; moreover, we here provide a retrospective analysis of the annual mortality by age groups and life stages in the years 2019 (pre-COVID-19), 2020 (before mass vaccination), and 2021 (after mass vaccination) among the whole Italian population in order to evaluate the death burden attributable to COVID-19.

## 2. Materials and Methods

### 2.1. Data Collection

During the state of national health emergency (expired on 31 March 2022), the Civil Defense Department daily released the aggregate data coming from the Higher Institute of Health, the Ministry of Health, the Italian Regions (Lombardia, Lazio, Campania, Veneto, Sicilia, Emilia-Romagna, Piemonte, Puglia, Toscana, Calabria, Sardegna, Liguria, Marche, Abruzzo, Friuli-Venezia Giulia, Umbria, Basilicata, Molise, and Valle d’Aosta), and the Independent Provinces (Trento and Bolzano) in order to inform the population about the pandemic situation in Italy. Among these data, on a daily basis, there were the number of contagions, performed swabs, hospitalizations in Intensive Care Units (ICU), non-ICU patients, and deaths [10].

By means of a team effort, we have collected all these data and elaborated the respective graphs, comparing the COVID-19 pandemic in Italy during the autumn–winter of 2020 (before mass vaccination) with the autumn–winter of 2021 (after mass vaccination).

### 2.2. Data Mining

In addition, we have consulted the database of the National Institute of Statistics [11] and extracted the total number of annual deaths in the years 2019 (pre-COVID-19), 2020 (before mass vaccination), and 2021 (after mass vaccination), broken down into gender and age groups by years (<4; 5–9; 10–14; 15–19; 20–24; 25–29; 30–34; 35–39; 40–44; 45–49; 50–54; 55–59; 60–64; 65–69; 70–74; 75–79; 80–84; 85–89; 90–94; >95), in order to evaluate the mortality burden attributable to COVID-19.

## 3. Results

### 3.1. COVID-19 Deaths

In the autumn–winter of 2020, there were 68,934 deaths due to COVID-19, of which there were 33,092 in autumn (22 September 2020–20 December 2020) and 35,842 in winter (21 December 2020–20 March 2021), while in the autumn–winter of 2021, there were 27,365 deaths, of which there were 5359 in autumn (22 September 2021–20 December 2021) and 22,006 in winter (21 December 2021–20 March 2022) (Table 1).

**Figure 1:**
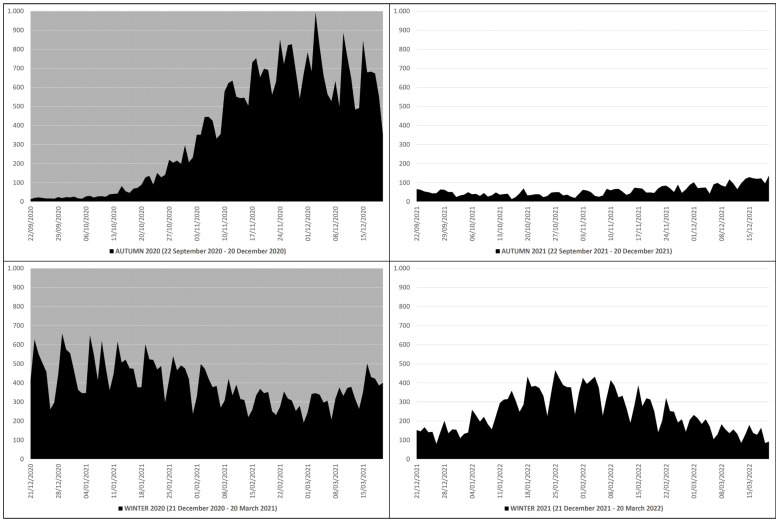
COVID-19 deaths in Italy during the autumn–winter of 2020 (22 September 2020–20 March 2021) and the autumn–winter of 2021 (22 September 2021–20 March 2022) collected in black graphs: the top left panel with gray background refers to the autumn of 2020 (22 September 2020–20 December 2020), the bottom left panel with gray background refers to the winter of 2020 (21 December 2020–20 March 2021), the top right panel with white background refers to the autumn of 2021 (22 September 2021–20 December 2021), and the bottom right panel with white background refers to the winter of 2021 (21 December 2021–20 March 2022) [*X* axis: days; *Y* axis: number of deaths].

This means that in the autumn–winter of 2020 (before mass vaccination), there were 41,569 more deaths than in the autumn–winter of 2021 (after mass vaccination), which equals ≈2.51 times more; in practice, from the autumn–winter of 2020 (before mass vaccination) to the autumn–winter of 2021 (after mass vaccination), there was a drop in deaths of ≈251%. The peak of deaths in the autumn–winter of 2020 (n. 993) occurred on 3 December 2020, while in the autumn–winter of 2021 (n. 468), it occurred on 25 January 2022 (Figure 1).

### 3.2. COVID-19 ICU Hospitalizations

**Figure 2:**
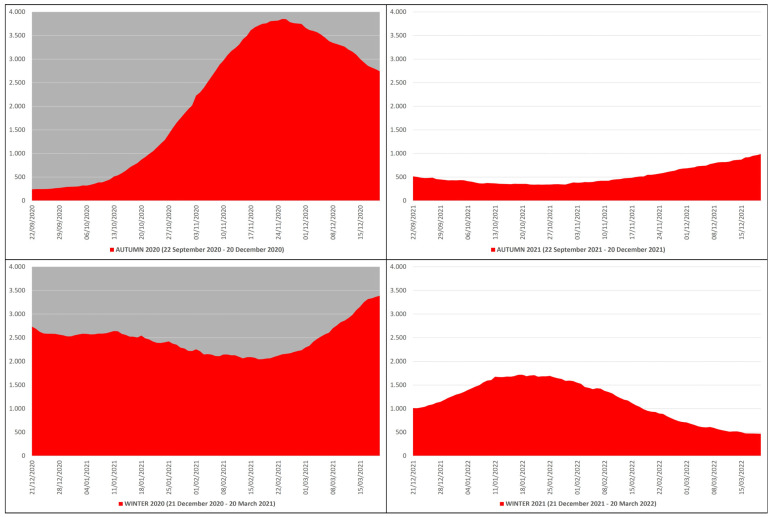
COVID-19 ICU hospitalizations in Italy during the autumn–winter of 2020 (22 September 2020–20 March 2021) and the autumn–winter of 2021 (22 September 2021–20 March 2022) collected in red graphs: the top left panel with gray background refers to the autumn of 2020 (22 September 2020–20 December 2020), the bottom left panel with gray background refers to the winter 2020 (21 December 2020–20 March 2021), the top right panel with white background refers to the autumn of 2021 (22 September 2021–20 December 2021), and the bottom right panel with white background refers to the winter of 2021 (21 December 2021–20 March 2022) [*X* axis: days; *Y* axis: number of ICU hospitalizations].

In the autumn–winter of 2020, the daily ICU hospitalizations due to COVID-19 averaged 2281, with an autumn average of 2091, a winter average of 2470, and a peak of 3848 ICU patients on 25 November 2020, while in the autumn–winter of 2021, the daily ICU hospitalizations averaged 843, with an autumn average of 522, a winter average of 1163, and a peak of 1717 ICU patients on 17 January 2022 (Figure 2). This means that in the autumn–winter of 2020 (before mass vaccination), the daily ICU hospitalization rate was ≈224% higher than in the autumn–winter of 2021 (after mass vaccination); in practice, from the autumn–winter of 2020 (before mass vaccination) to the autumn–winter of 2021 (after mass vaccination), the rate fell ≈2.24 times.

### 3.3. COVID-19 Non-ICU Hospitalizations

**Figure 3:**
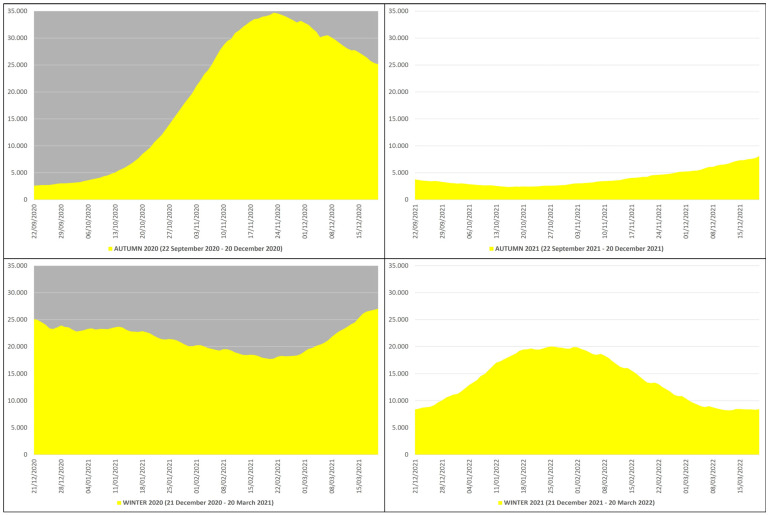
COVID-19 non-ICU hospitalizations in Italy during the autumn–winter of 2020 (22 September 2020–20 March 2021) and the autumn–winter of 2021 (22 September 2021–20 March 2022) collected in yellow graphs: the top left panel with gray background refers to the autumn of 2020 (22 September 2020–20 December 2020), the bottom left panel with gray background refers to the winter of 2020 (21 December 2020–20 March 2021), the top right panel with white background refers to the autumn of 2021 (22 September 2021–20 December 2021), and the bottom right panel with white background refers to the winter of 2021 (21 December 2021–20 March 2022) [*X* axis: days; *Y* axis: number of non-ICU hospitalizations].

In the autumn–winter of 2020, the daily non-ICU hospitalizations due to COVID-19 averaged 20,489, with an autumn average of 19,357, a winter average of 21,621, and a peak of 34,697 non-ICU patients on 23 November 2020, while in the autumn–winter of 2021, the daily non-ICU hospitalizations averaged 8977, with an autumn average of 4024, a winter average of 13,929, and a peak of 20,027 non-ICU patients on 25 January 2022 (Figure 3). This means that in the autumn–winter of 2020 (before mass vaccination), the daily non-ICU hospitalization rate was ≈228% higher than in the autumn–winter of 2021 (after mass vaccination); in practice, from the autumn–winter of 2020 (before mass vaccination) to the autumn–winter of 2021 (after mass vaccination), the rate fell ≈2.28 times.

### 3.4. COVID-19 Contagions

**Figure 4:**
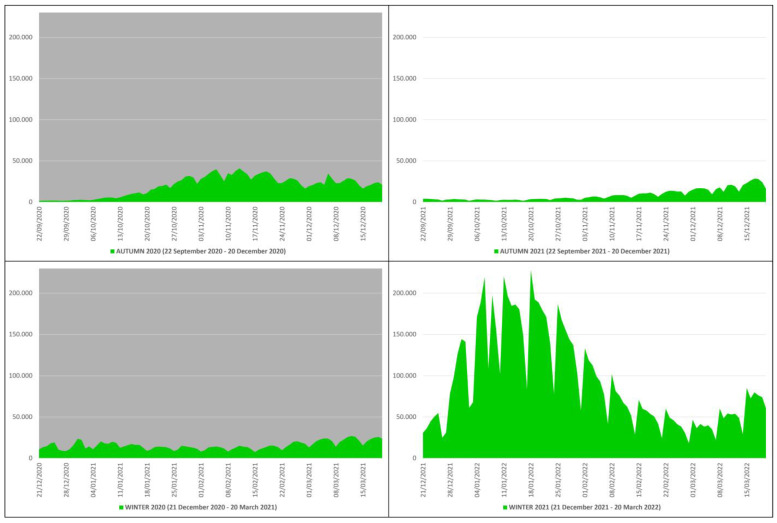
COVID-19 contagions in Italy during the autumn–winter of 2020 (22 September 2020–20 March 2021) and the autumn–winter of 2021 (22 September 2021–20 March 2022) collected in green graphs: the top left panel with gray background refers to the autumn of 2020 (22 September 2020–20 December 2020), the bottom left panel with gray background refers to the winter of 2020 (21 December 2020–20 March 2021), the top right panel with white background refers to the autumn of 2021 (22 September 2021–20 December 2021), and the bottom right panel with white background refers to the winter of 2021 (21 December 2021–20 March 2022) [*X* axis: days; *Y* axis: number of contagions].

In the autumn–winter of 2020, the number of COVID-19 contagions was 3,179,094, of which there were 1,774,864 in autumn and 1,404,230 in winter, while in the autumn–winter of 2021, there were 9,068,535, of which 762,613 were in autumn and 8,305,922 in winter (Table 1). This means that from the autumn–winter of 2020 (before mass vaccination) to the autumn–winter of 2021 (after mass vaccination), the COVID-19 contagions increased by 5,889,441 subjects, an increase of ≈2.85 times equal to ≈285%. The peak of contagions in the autumn–winter of 2020 (n. 40,902) occurred on 13 November 2020, while in the autumn–winter of 2021 (n. 228,179), it occurred on 18 January 2022 (Figure 4).

### 3.5. COVID-19 Swab Tests

**Figure 5:**
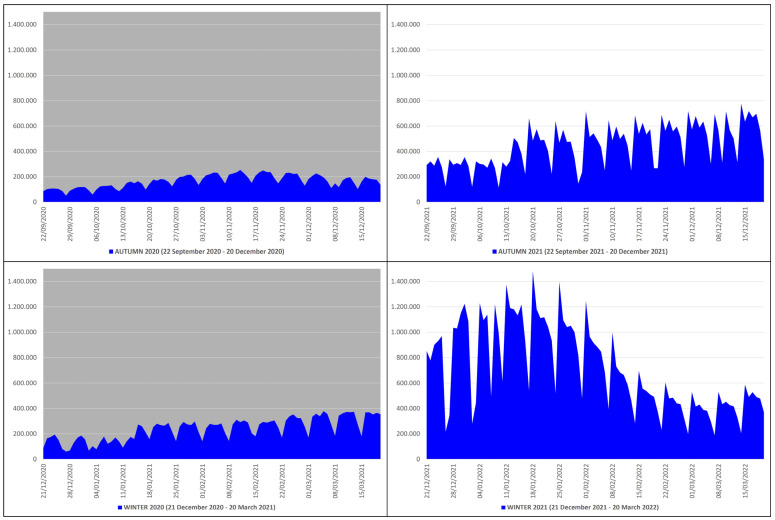
COVID-19 swab tests in Italy during the autumn–winter of 2020 (22 September 2020–20 March 2021) and the autumn–winter of 2021 (22 September 2021–20 March 2022) collected in blue graphs: the top left panel with gray background refers to the autumn of 2020 (22 September 2020–20 December 2020), the bottom left panel with gray background refers to the winter of 2020 (21 December 2020–20 March 2021), the top right panel with white background refers to the autumn of 2021 (22 September 2021–20 December 2021), and the bottom right panel with white background refers to the winter of 2021 (21 December 2021–20 March 2022) [*X* axis: days; *Y* axis: number of swabs].

In the autumn–winter of 2020, the number of swabs performed to detect COVID-19 was 36,396,019, of which 14,878,410 were in autumn and 21,517,609 in winter, while in the autumn–winter of 2021, there were 105,553,802, of which 40,618,200 were in autumn and 64,935,602 in winter (Table 1). This means that from the autumn–winter of 2020 (before mass vaccination) to the autumn–winter of 2021 (after mass vaccination), the swab tests increased by 69,157,783 units, an increase of ≈2.90 times equal to ≈290%. The peak of performed tests in the autumn–winter of 2020 (n. 378,463) occurred on 5 March 2021, while in the autumn–winter of 2021 (n. 1,481,349), it occurred on 18 January 2022 (Figure 5).

### 3.6. Mortality Burden

By analyzing the mortality data in Italy, it has emerged that in 2019 (pre-COVID-19), 634,417 people died, of which 303,652 were males and 330,764 females, while in 2020 (before mass vaccination), there were 740,317 deaths, 359,418 males and 380,899 females (Table 2). In the year 2019, the Italian population consisted of 60.32 million people, then progressively decreased to 59.26 million in 2020 and 58.98 million in 2021 [4,5,6,7]; the national mortality rate therefore increased from ≈10.5‰ for 2019 to ≈12.5‰ for 2020.

In practice, from 2019 to 2020, there was an excess of mortality quantifiable in 105,900 more deaths attributable to COVID-19, as the only significant variable changed between the two years. This death burden represents the ≈14.3% of the total. If we then apply the standardized age disaggregation groups recommended for data analysis by life stages [12], 103,183 out of 105,900 deaths occurred in older adults (≥60 years), which is equivalent to ≈97.4%. In 2021 (after mass vaccination), there were 701,346 deaths, of which 340,210 were males and 361,136 females (Table 2), with a national mortality rate of ≈11.8‰, lower than 2020 but higher than 2019. Comparing 2019 (pre-COVID-19) to 2021 (after mass vaccination), an excess of mortality is confirmed, quantifiable in 66,929 more deaths, a value that is, however, lower than in 2020 (before mass vaccination). Among adults (25–59 years), in 2019, 2020, and 2021, there were 42,163, 45,114, and 45,531 deaths, respectively (Table 2). As can be seen from this, COVID-19 has caused a mortality excess of 2951 more deaths (2125 males and 826 females) from 2019 (pre-COVID-19) to 2020 (before mass vaccination) in this life stage, of which 2807 were over 50, i.e., ≈95.1%. Among young adults (20–24 years), older adolescents (15–19 years), young adolescents (10–14 years), older children (5–9 years), young children (1–4 years), post-neonatal infants (28–364 days), late neonates (7–27 days), and early neonates (0–6 days), in 2019, 2020, and 2021 there were 2962, 2728, and 2820 deaths, respectively (Table 2). Therefore, in 2019 (pre-COVID-19), there were 238 and 142 more deaths than in the years 2020 and 2021, both impacted by COVID-19.

Surprisingly, the excess of mortality under the age of 60 continued to grow by 509 subjects (316 males and 193 females) from 2020 (before mass vaccination) to 2021, the unprecedented year of the mass vaccination campaign. If we further restrict the field of analysis to subjects under the age of 40, it emerges that in 2019, 2020, and 2021, there were 7103, 6808, and 7165 deaths, respectively (Table 2). Therefore, during 2020 (before mass vaccination), there were 295 fewer deaths (246 males and 49 females) than in 2019 (pre-COVID-19), equivalent to ≈4.2% less, while during 2021 (after mass vaccination), there were 357 more deaths (247 males and 110 females) than in 2020 (before mass vaccination) and 62 more deaths (1 male and 61 females) than in 2019 (pre-COVID-19), equivalent to ≈5.2% and ≈0.9% more.

## 4. Discussion

COVID-19 was first confirmed to have spread to Italy on 31 January 2020, when two Chinese tourists in Rome tested positive for the virus; one week later, an Italian man, repatriated to Italy from the city of Wuhan, was hospitalized and ascertained as the third case. From that day, Italy was overwhelmed by four waves of the pandemic [13], and 2020 became the year with the highest number of deaths since 1945, when Italy was fighting in World War II on its soil. The four waves are timed as follows: the first wave occurred from February to May 2020; the second wave occurred from October to December 2020; the third wave occurred from January to May 2021; the fourth wave occurred from November 2021 to March 2022 [13]. All the waves’ peaks coincide with the coldest seasons in Italy; in fact, SARS-CoV-2 virulence has been found to be maximum below 10 °C and 40 kJ/m^2^ [9]. This behavior towards environmental temperature and solar ultraviolet radiation is quite similar to that of other viral respiratory infections, in primis influenza [14]. Our study has therefore been focused on the coldest seasons of the year in Italy, i.e., the autumn–winter periods. The Italian government faced the aforementioned waves with gradually decreasing lockdown restrictions thanks to the progressive availability of masks, swabs, antivirals, and above all, starting from the beginning of 2021, new generation vaccines, such as adenovirus vector-based or modRNA vaccines [1]. Among the former, there are Vaxzevria^®^ by AstraZeneca and Jcovden^®^ by Janssen, while among the latter are Comirnaty^®^ by Pfizer/BioNTech and Spikevax^®^ by Moderna [15].

By virtue of this, health policies were adopted that strongly encouraged free-of-charge mass vaccination, so as to avoid differences between low, medium, and high income social groups and to increase the number of people vaccinated. In the period of time taken into consideration in our study, approximately 135 million doses of COVID-19 vaccines were administered, of which 65.2% were Comirnaty^®^, 24.7% Spikevax^®^, 9.0% Vaxzevria^®^, and 1.1% Jcovden^®^ [15]. A coverage of the population over 12 equal to 90.25% and 84.89% was achieved with regard to the primary vaccination cycle (two doses) and the booster (three doses), respectively; the percentage of partially vaccinated subjects (one dose) reached 91.72% [3].

By comparing the trend of COVID-19 pandemic in Italy during the autumn–winter of 2020, which encompasses the second and third waves, to the autumn–winter of 2021, which includes the fourth wave, we have found that the contagions increased by ≈285%, against a ≈290% increase in the swab tests. The absolute peak of contagions coincided with 18 January 2022, the very day on which the highest number of swabs was performed. The mean positivity rate therefore passed from ≈8.74% before mass vaccination to ≈8.59% after mass vaccination. All this means that, from one side, COVID-19 vaccines are not able to stop the contagions, but, from the other side, they have been able to reduce the mean positivity rate by ≈0.15% despite the progressive easing of lockdown, which remains the most effective health policy measure to block contagions’ surge in an emergency phase when specific vaccines are not yet available, together with wearing mask indoors (preferably with filtering face pieces), practicing hand hygiene (with soap or hydroalcoholic solution), and keeping interpersonal social distancing (at least 1 m).

Moreover, we have found that, from the autumn–winter of 2020 to the autumn–winter 2021, the mass vaccination campaign, without precedent in Italy as in the rest of the world, allowed a ≈251% abatement in COVID-19 deaths and a reduction of ≈224% and ≈228% in daily ICU hospitalizations and daily non-ICU hospitalizations due to COVID-19, respectively. These results are impressive and definitively confirm the usefulness of anti-SARS-CoV-2 vaccination in reducing the number of deaths and hospitalizations on a large scale; this is reflected in a significant lowering of pressure on hospitals and, in particular, on ICU departments, thus safeguarding the health service in the public interest.

Going into the details of COVID-19 deaths, in 2020, there was a mortality excess of ≈14.3% quantifiable in 105,900 more deaths compared to 2019, the pre-COVID-19 year; 103,183 out of 105,900 deaths occurred in older adults, which is equivalent to ≈97.4%. Therefore, COVID-19 is confirmed as a potentially life-threating disease in older adults, who are the most at risk and a vulnerable segment of population, given their immunosenescence. Immunosenescence is the gradual deterioration of the immune system, determined by natural age advancement, and represents a contributory factor to the increased frequency of mortality and morbidity among the elderly; both the host’s capacity to respond to infections and the development of long-term immune memory are affected by immunosenescence [16]. Immunosenescence may also explain the mortality excess of 2807 more deaths from 2019 to 2020 found by us among adults over 50, the segment of the population just below older adults. Another possible explanation lies in the concept of inflammaging, which represents a further risk factor for mortality and morbidity with advancing age [17]. Unlike immunosenescence, inflammaging is a chronic low-grade inflammation in the absence of overt infection that occurs in aging, and may contribute to the clinical manifestations of other age-related pathologies; it is probably due to a loss of power of adaptive immunity and to a loss of control over systemic inflammation resulting in the release of pro-inflammatory cytokines, such as interleukin 6, the well-known molecule involved in those serious forms of COVID-19 characterized by «cytokine storm» [18,19,20,21].

Surprisingly, from our real-world study on a very large population, the picture has completely changed by varying the population segment taken into consideration. In fact, it emerged that among people under the age of 40 in the years 2019, 2020, and 2021 there were 7103, 6808, and 7165 deaths, respectively. Therefore, during 2020 (before mass vaccination), there were 295 fewer deaths than in 2019 (pre-COVID-19), equivalent to ≈4.2% less, while during 2021 (after mass vaccination), there were 357 more deaths than in 2020 (before mass vaccination), equivalent to ≈5.2% more. COVID-19 is thus confirmed as a potentially lethal illness in older adults, but not in young people; indeed, from our study results, the benefit–risk balance of COVID-19 vaccination decreases in people under the age of 40. Scientific proof of the possible complications, even fatal, in young people due to COVID-19 vaccination lies in vaccine-induced immune thrombotic thrombocytopenia (VITT), a new blood clotting syndrome initially described in association with adenovirus vector-based COVID-19 vaccines [22,23,24]. VITT is characterized by both arterial and venous thrombotic events in unusual sites, such as splanchnic veins and cerebral venous sinus, accompanied by low platelets count (<150 × 10^3^/mm^3^), markedly elevated D-dimer (>4 times upper limit of normal), and positive Platelet Factor 4 (PF4) Heparin-Induced Thrombocytopenia (HIT) Enzyme-Linked Immuno-Sorbent Assay (ELISA), 4–42 days after vaccine administration; pulmonary embolism and disseminated intravascular coagulation may also occur [25]. Among the lab tests available today for the detection of pathological platelet-activating antibodies against PF4, that is to say, VITT triggers, HIT ELISA is the one with the greatest validity and reliability and with the most appropriate sensitivity, reaching around 95% [25]. Although these adverse events are very rare, they exceeded what would be expected in the general population [26].

Like all ribonucleic acid viruses, SARS-CoV-2 shows a high mutation rate which has led to the emergence of variants [27]. During the autumn–winter of 2020, the dominant SARS-CoV-2 strain was the Alpha variant (≈75% of cases), while during the autumn–winter of 2021, at first, the Delta variant (≈90% of cases), and subsequently, the Omicron variant (≈100% of cases) emerged [28]. Here, we have disclosed that, from the autumn–winter of 2020 to the autumn–winter of 2021, the mass vaccination allowed a ≈251% abatement in COVID-19 deaths. In Italy, this mass vaccination campaign was implemented with vaccines based on the Wuhan strain; therefore, we can argue that COVID-19 vaccines, developed by exploiting the non-predominant ancestral antigen, conferred cross-protection against the SARS-CoV-2 variants that would emerge soon. However, the available data do not allow us to correlate every single death with the variant involved in the death, and so it is not possible for us to calculate the case fatality rate (CFR), a first limitation of our study. Despite this, a recent global meta-analysis has estimated that the deadliest SARS-CoV-2 variant was the Beta variant (CFR: 4.19%), followed by the Gamma variant (CFR: 3.60%), the Alpha variant (CFR: 2.62%), the Delta variant (CFR: 2.01%), and the Omicron variant (CFR: 0.70%) [29]. The Omicron variant appears much less deadly than the Alpha and Delta variants, presumably due to immunity from vaccination or previous infection and to a better adaptation of the virus strain towards the human host [30], all factors to consider when explaining the decreased mortality from the autumn–winter of 2020 to the autumn–winter of 2021. Based on our research, from 2020 (before mass vaccination) to 2021 (after mass vaccination), the death burden in older adults was in fact decreased by 39,480 subjects, an extraordinary gain in terms of life attributable to both variables at play, namely acquired immunity and less-virulent strains.

A second limitation of our study is represented by the fact that the available data do not allow us to know in what proportion the deaths and ICU hospitalizations in the autumn–winter of 2021 occurred among fully vaccinated (90.25% of the population over 12), partially vaccinated (91.72%), and unvaccinated (8.28%) subjects, while a third limitation lies in the impossibility of knowing the type of vaccine administered to the fully or partially vaccinated, whether viral vector-based or modRNA. Moreover, a fourth limitation of our study is represented by the unavailability of data on a large scale regarding any other comorbidities of the deceased patients (e.g., immune deficiencies).

## 5. Conclusions

In conclusion, supported by a real-world nationwide study, just over four years after the COVID-19 outbreak, we can now state the following: (I) COVID-19 is a potential life-threatening disease mainly in older adults, as they are more vulnerable because of inherent immunosenescence and inflammaging; (II) vaccination appears useful for reducing deaths and ICU/non-ICU hospitalizations due to COVID-19 in this segment of population, at the same time reducing the pressure on hospitals in the public interest; (III) vaccine immunization, immunity from previous infection, and the CFR of SARS-CoV-2 variants compete in reducing deaths; (IV) COVID-19 vaccination shows a favorable benefit–risk ratio in older adults, while the balance steps down under the age of 40 from our study results; (V) subjects under the age of 40 should be therefore exempt from any mandatory vaccination, which should be left to their personal choice; (VI) an effective vaccination campaign against COVID-19 should be primarily aimed at people over 60 and at patients of any age with immune deficits, and secondly at people over 50; (VII) in the hypothetical event of a future threat by a novel coronavirus strain, lockdown remains the most effective measure of health policy to stop the contagions, when specific vaccines are not yet available, together with wearing mask indoors, practicing hand hygiene, and keeping interpersonal social distancing.

## Figures and Tables

**Table 1:** Number of total COVID-19 deaths, contagions and swab tests and of daily ICU and non-ICU patients during the autumn–winter of 2020 (22 September 2020–20 March 2021) and the autumn–winter of 2021 (22 September 2021–20 March 2022).

Autumn–Winter	2020	2021
COVID-19 deaths	68,934	27,365
COVID-19 contagions	3,179,094	9,068,535
COVID-19 swab tests	36,396,019	105,553,802
COVID-19 daily ICU	2281	843
COVID-19 daily non-ICU	20,489	8977

**Table 2:** Total mortality in Italy for the years 2019 (pre-COVID-19), 2020 (before mass vaccination), and 2021 (after mass vaccination), broken down into gender (♂: male; ♀: female) and age groups by years (<4; 5–9; 10–14; 15–19; 20–24; 25–29; 30–34; 35–39; 40–44; 45–49; 50–54; 55–59; 60–64; 65–69; 70–74; 75–79; 80–84; 85–89; 90–94; >95).

Age	♂ 2019 ♀		♂ 2020 ♀		♂ 2021 ♀	
**<4**	696	543	628	507	601	500
**5‒9**	80	69	78	71	80	70
**10‒14**	122	88	133	85	112	115
**15‒19**	408	142	327	162	385	188
**20‒24**	623	191	550	187	583	186
**25‒29**	718	277	654	242	727	285
**30‒34**	862	369	821	406	909	407
**35‒39**	1199	716	1271	686	1312	705
**40‒44**	2336	1407	2318	1343	2311	1331
**45‒49**	4119	2556	4276	2686	4284	2647
**50‒54**	6903	4202	7491	4548	7401	4529
**55‒59**	10,216	6283	11,647	6725	11,805	6878
**60‒64**	14,214	8705	16,899	9481	16,640	9659
**65‒69**	20,561	12,164	24,256	13,432	23,543	13,872
**70‒74**	30,782	19,677	38,784	23,189	36,501	23,225
**75‒79**	42,040	30,800	48,980	34,224	45,445	32,876
**80‒84**	56,739	52,779	69,530	61,826	64,164	58,646
**85‒89**	60,155	76,445	70,604	87,884	65,629	81,356
**90‒94**	37,829	72,671	45,145	85,524	43,065	78,909
**>95**	13,050	40,681	15,026	47,691	14,713	44,752
	**303,652**	**330,765**	**359,418**	**380,899**	**340,210**	**361,136**

## Data Availability

Publicly available datasets were analyzed in this study; further requests for data should be addressed to the corresponding author due to legal reason.
